# Evaluating the S100β, GFAP, IL-6, and Oxidative Stress Markers in Traumatic Brain Injury

**DOI:** 10.7759/cureus.90329

**Published:** 2025-08-17

**Authors:** Adeel Ur Rehman, Anees Ahmed, Tayyaba Ali, Mubariz Ali, Nasir Jamil, Farooq Ahmad Malik, Imtiaz Mustafa

**Affiliations:** 1 Neurosurgery, Punjab Institute of Neurosciences, Lahore, PAK; 2 Physiology, Liaquat Institute of Medical and Health Sciences, Thatta, PAK; 3 Histopathology, Islamabad Diagnostic Centre, Islamabad, PAK; 4 Surgery, Combined Military Hospital (CMH), Kharian, PAK; 5 Physiology, Liaquat College of Medicine and Dentistry, Darul Sehat Hospital, Karachi, PAK; 6 Biochemistry, Dera Ghazi Khan Medical College, Dera Ghazi Khan, PAK; 7 Institute of Molecular Biology and Biotechnology, The University of Lahore, Lahore, PAK

**Keywords:** gfap, il-6, oxidative stress, s100β, traumatic brain injury

## Abstract

Background

Traumatic brain injury (TBI) is a leading cause of neurological morbidity and mortality worldwide. Secondary injury mechanisms such as neuroinflammation and oxidative stress play a pivotal role in the progression of neuronal damage. Biomarkers, including S100β, glial fibrillary acidic protein (GFAP), interleukin-6 (IL-6), and oxidative stress parameters, can reflect the severity and progression of TBI, and their evaluation may assist in clinical decision-making.

Objective

This study aims to evaluate the serum levels of S100β, GFAP, IL-6, and oxidative stress markers (malondialdehyde (MDA), superoxide dismutase (SOD), and catalase) in patients with TBI compared to healthy controls.

Methods

This case-control study was conducted at The University of Lahore from July to December 2024. A total of 96 participants were enrolled, including 48 TBI patients and 48 age- and sex-matched healthy controls. Blood samples were collected within 24 hours of injury in TBI patients. Serum concentrations of S100β, GFAP, and IL-6 were measured using ELISA (enzyme-linked immunosorbent assay), while MDA, SOD, and catalase levels were assessed using standard biochemical assays. Data were analyzed using IBM SPSS Statistics for Windows, Version 26 (Released 2019; IBM Corp., Armonk, New York). Independent sample t-tests were used for group comparisons, and Pearson's correlation was applied to examine biomarker relationships.

Results

TBI patients exhibited significantly higher serum levels of S100β (1.92 ± 0.46 vs. 0.61 ± 0.18 ng/mL), GFAP (3.45 ± 0.71 vs. 1.12 ± 0.33 ng/mL), IL-6 (82.6 ± 18.4 vs. 19.5 ± 7.6 pg/mL), and MDA (5.12 ± 1.03 vs. 2.23 ± 0.66 µmol/L) compared to controls (p < 0.001 for all). SOD and catalase activities were significantly reduced in TBI patients (p < 0.001). S100β and GFAP correlated positively with IL-6 and MDA and negatively with SOD and catalase.

Conclusion

TBI is associated with elevated neural injury, inflammation, and oxidative stress biomarkers. These findings highlight the diagnostic and prognostic potential of these markers in TBI management.

## Introduction

Traumatic brain injury (TBI) is a leading cause of mortality and long-term disability worldwide, particularly among young adults. It is characterized by physical damage to the brain that may temporarily or permanently impair cognitive, behavioral, and neurological functions [1]. In the United States alone, TBI is responsible for approximately 1.5 million hospitalizations and fatalities annually. Based on the source and nature of mechanical forces, TBI is classified into three categories: ballistic, blunt, and blast-related injuries. Ballistic injuries occur when a penetrating object breaches the skull and enters the brain, with the projectile either lodging within or exiting through the opposite side, often accompanied by brain tissue displacement depending on its kinetic energy at the point of impact [2]. In blunt injuries, the head collides with a moving or stationary object, transmitting localized directional force that results in rotational or translational movement of the brain. Blast injuries combine features of both ballistic and blunt trauma and are typically seen in combat or explosion scenarios [3].

TBI severity is commonly assessed using the Glasgow Coma Scale (GCS), a standardized tool for evaluating consciousness. The GCS includes three components: eye opening, verbal response, and motor response. A total score of 13-15 indicates mild TBI, 9-12 denotes moderate injury, and 3-8 signifies severe TBI [4].

The pathophysiology of TBI involves two distinct mechanisms: primary and secondary brain injury. Primary injury results from mechanical forces at the time of trauma and includes irreversible damage such as skull fractures, axonal shearing, contusions, blood vessel rupture, and intracranial hemorrhages [5,6]. In contrast, secondary brain injury develops over time and is influenced by a cascade of biochemical and molecular events, including increased intracranial pressure, blood-brain barrier (BBB) disruption, cerebral edema, ischemia, hypoxia, and neuroinflammation [7,8]. These delayed processes may worsen neurological outcomes but are potentially modifiable with therapeutic interventions [9].

Oxidative stress is a critical component of secondary injury and has been extensively reported to exacerbate TBI-induced cellular damage [10]. It results from an imbalance between reactive oxygen species (ROS) or reactive nitrogen species (RNS) and the brain's intrinsic antioxidant defenses, such as superoxide dismutase (SOD), glutathione peroxidase, and catalase. Elevated levels of ROS/RNS promote lipid peroxidation, protein oxidation, DNA fragmentation, mitochondrial dysfunction, and activation of apoptotic pathways [11]. These pathophysiological processes contribute significantly to neuroinflammation, BBB disruption, and cognitive impairment following TBI [9].

The role of specific biomarkers, particularly S100β, glial fibrillary acidic protein (GFAP), and interleukin-6 (IL-6), in reflecting the extent of neuronal injury, glial activation, and systemic inflammation is well recognized [12,13]. Simultaneously, oxidative stress markers such as malondialdehyde (MDA), SOD, and catalase offer valuable insight into the redox status and mitochondrial dysfunction in TBI. Given the critical role of these markers in TBI progression and prognosis, their evaluation may help guide early diagnosis, monitor disease severity, and inform therapeutic strategies. Therefore, the objective of this study was to assess serum levels of S100β, GFAP, IL-6, and oxidative stress markers (MDA, SOD, and catalase) in patients with TBI compared to healthy controls.

## Materials and methods

This observational, case-control study was conducted at The University of Lahore, Lahore, Pakistan, over a period of six months, from July 1, 2024, to December 31, 2024. The study was designed to evaluate serum levels of S100β, GFAP, IL-6, and oxidative stress markers in patients with TBI, in comparison to age- and sex-matched healthy controls.

A total sample size of 96 participants was calculated using the OpenEpi software (https://www.openepi.com), based on a 95% confidence interval, 80% power, and a medium effect size (Cohen's d = 0.5) for biomarker differences between groups, with an expected ratio of 1:1 between cases and controls. Accordingly, 48 patients with clinically diagnosed TBI and 48 healthy individuals were recruited.

Participants were selected using a non-probability consecutive sampling technique. Inclusion criteria for the TBI group included adult patients aged 18 to 60 years, admitted to the emergency department within 24 hours of sustaining a closed head injury, and confirmed TBI diagnosis via computed tomography (CT) scan showing specific radiological findings such as intracranial hemorrhage (epidural, subdural, or intracerebral), contusions, diffuse axonal injury, or brain edema, graded according to the Marshall CT Classification. Hematoma volume was calculated using the ABC/2 method for patients with intracranial bleeds. Patients with polytrauma, known neurodegenerative disorders, malignancies, chronic inflammatory diseases, or prior brain surgeries were excluded. CT severity grades and hematoma volumes were recorded for all patients and correlated with biomarker levels (S100β, GFAP, IL-6, MDA, SOD, and catalase) during data analysis to explore relationships between radiological severity and biochemical parameters. The control group consisted of healthy volunteers with no history of neurological disease or systemic illness, matched for age and sex.

Following written informed consent, venous blood samples were collected from all participants. For TBI patients, samples were obtained within 24 hours of injury. Serum was separated by centrifugation and stored at -80°C until analysis.

S100β and GFAP concentrations were determined using commercially available human ELISA (enzyme-linked immunosorbent assay) kits (Bioassay Technology Laboratory, Shanghai, China) with validated sensitivity and intra- and inter-assay precision. IL-6 levels were quantified using a high-sensitivity sandwich ELISA kit (Thermo Fisher Scientific, Waltham, MA, USA), according to the manufacturer's protocol.

Oxidative stress was assessed through measurement of serum MDA levels as a marker of lipid peroxidation and enzymatic antioxidant activity, including SOD and catalase. MDA was quantified using the thiobarbituric acid reactive substances (TBARS) assay, while SOD and catalase activities were measured using standardized colorimetric methods (Cayman Chemical, Ann Arbor, MI, USA) as previously reported by Mustafa et al. [14].

Data were recorded and analyzed using IBM SPSS Statistics for Windows, Version 26 (Released 2019; IBM Corp., Armonk, New York). Continuous variables were expressed as mean ± standard deviation (SD) and compared between groups using independent sample t-tests for normally distributed data and Mann-Whitney U tests for non-parametric data. Categorical variables were presented as frequencies and percentages and analyzed using the chi-square test. Pearson or Spearman correlation analysis was used to evaluate relationships between biomarkers. A p-value of <0.05 was considered statistically significant.

Ethical approval for this study was obtained from the Institutional Ethical Review Committee of The University of Lahore (Ref number: IMBB/BBBC/24/156/B) prior to the commencement of data collection. All procedures were conducted in accordance with the principles outlined in the Declaration of Helsinki.

## Results

A total of 96 participants were enrolled in the study, comprising 48 patients with TBI (TBI group) and 48 age- and sex-matched healthy individuals (control group). The mean age of the TBI group was 36.2 ± 11.3 years, while that of the control group was 34.8 ± 10.7 years (p = 0.456). Both groups had a male-to-female ratio of 3:1.

Biomarkers of neural injury and inflammation (S100β, IL-6, and GFAP)

Serum concentrations of S100β and GFAP were significantly elevated in the TBI group compared to the control group. The mean serum S100β level in the TBI group was 1.92 ± 0.46 ng/mL, significantly higher than 0.61 ± 0.18 ng/mL in the control group (p < 0.001). Similarly, GFAP levels were markedly increased in TBI patients (3.45 ± 0.71 ng/mL) compared to controls (1.12 ± 0.33 ng/mL, p < 0.001). IL-6 levels were significantly higher in TBI patients compared to those in the control group. The TBI group had a mean IL-6 concentration of 82.6 ± 18.4 pg/mL, while the control group exhibited a significantly lower mean of 19.5 ± 7.6 pg/mL (p < 0.001) (Table 1).

**Table 1:** Comparison of Serum S100β, GFAP, and IL-6 Levels Between the TBI and Control Groups An independent sample t-test was applied. Values are presented as mean ± SD. The degree of freedom was 94. Values are presented as mean ± SD. S100β: S100 calcium-binding protein β, GFAP: Glial fibrillary acidic protein, IL-6: Interleukin-6, TBI: Traumatic brain injury, t: independent samples t-test statistic, Cohen's d: effect size.

Biomarker	TBI Group (n = 48)	Control Group (n = 48)	p-value	t statistics	Effect Size (Cohen's d)
S100β (ng/mL)	1.92 ± 0.46	0.61 ± 0.18	< 0.001	18.13	3.73
GFAP (ng/mL)	3.45 ± 0.71	1.12 ± 0.33	< 0.001	19.64	3.99
IL-6 (pg/mL)	82.6 ± 18.4	19.5 ± 7.6	< 0.001	20.83	4.23

Oxidative stress parameters (MDA, SOD, and catalase)

Assessment of oxidative stress markers revealed significantly elevated MDA levels and reduced antioxidant enzyme activities (SOD and catalase) in the TBI group. The mean serum MDA levels in the TBI group were 5.12 ± 1.03 µmol/L, compared to 2.23 ± 0.66 µmol/L in the control group (p < 0.001). Conversely, the SOD activity was significantly reduced in TBI patients (1.68 ± 0.42 U/g FW) versus controls (2.84 ± 0.55 U/g FW, p < 0.001), as was catalase activity (3.45 ± 0.79 U/g FW in TBI vs. 5.91 ± 1.02 U/g FW in controls, p < 0.001) (Table 2).

**Table 2:** Oxidative Stress Markers in the TBI and Control Groups An independent sample t-test was applied. Values are presented as mean ± SD. The degree of freedom was 94. TBI: Traumatic brain injury, MDA: Malondialdehyde, SOD: Superoxide dismutase, FW: Fresh weight, U/g FW: Units per gram of fresh weight, µmol/L: Micromoles per liter, t: Independent samples t-test statistic, df: Degrees of freedom, Cohen's d: Effect size.

Parameter	TBI Group (n = 48)	Control Group (n = 48)	p-value	t statistics	Cohen's d
MDA (µmol/L)	5.12 ± 1.03	2.23 ± 0.66	< 0.001	17.14	3.51
SOD (U/g FW)	1.68 ± 0.42	2.84 ± 0.55	< 0.001	−12.12	2.49
Catalase (U/g FW)	3.45 ± 0.79	5.91 ± 1.02	< 0.001	−13.93	2.84

Correlation analysis between biomarkers in the TBI group

A Pearson correlation analysis was performed to assess associations between neural injury biomarkers and oxidative stress markers in TBI patients. S100β showed a significant positive correlation with MDA levels (r = 0.642, p < 0.001) and IL-6 (r = 0.583, p < 0.001) and a negative correlation with SOD (r = -0.461, p = 0.002). GFAP also positively correlated with MDA (r = 0.604, p < 0.001) and IL-6 (r = 0.537, p < 0.001) and negatively with catalase (r = -0.491, p = 0.001) (Table 3).

**Table 3:** Correlation of Neural Injury Biomarkers with Inflammatory and Oxidative Stress Markers in the TBI Group Pearson correlation analysis was applied. The degree of freedom was 46. S100β: S100 calcium-binding protein β, GFAP: Glial fibrillary acidic protein, IL-6: Interleukin-6, MDA: Malondialdehyde, SOD: Superoxide dismutase, Pearson r: Pearson correlation coefficient.

Correlation Pair	Pearson r	p-value
S100β vs. MDA	0.642	< 0.001
S100β vs. IL-6	0.583	< 0.001
S100β vs. SOD	-0.461	0.002
GFAP vs. MDA	0.604	< 0.001
GFAP vs. IL-6	0.537	< 0.001
GFAP vs. Catalase	-0.491	0.001

## Discussion

This study evaluated the serum levels of neural injury biomarkers (S100β and GFAP), the inflammatory cytokine IL-6, and oxidative stress indicators (MDA, SOD, and catalase) in patients with TBI compared to healthy controls. The findings strongly support the involvement of oxidative stress and neuroinflammation in TBI pathogenesis, consistent with mechanisms previously established in experimental and clinical studies.

Our results demonstrate significantly elevated serum levels of S100β (1.92 ± 0.46 ng/mL) and GFAP (3.45 ± 0.71 ng/mL) in TBI patients compared to controls (p < 0.001 for both). These findings align with previous research indicating that S100β, a glial-specific calcium-binding protein, and GFAP, an intermediate filament protein expressed in astrocytes, are reliable biomarkers of neural injury and BBB disruption [15]. Abdul-Muneer et al. [15] reported elevated S100β in animal models following blast-induced TBI, attributing the rise to astrocytic damage and compromised BBB integrity. The increased permeability of the BBB permits the leakage of brain-specific proteins into the systemic circulation, which is observed within hours of injury onset.

GFAP elevation similarly reflects astrocytic reactivity and structural brain damage. Its utility in distinguishing TBI severity has been emphasized in both preclinical and clinical contexts. The positive correlation between S100β and GFAP with oxidative stress (MDA) and IL-6 in our data further supports their pathophysiological relevance, extending beyond their role as diagnostic markers.

Serum IL-6 levels were markedly higher in the TBI group (82.6 ± 18.4 pg/mL) compared to the control group (19.5 ± 7.6 pg/mL), suggesting a robust neuroinflammatory response. These results are consistent with the literature, which shows that IL-6, alongside IL-1β and TNF-α, is rapidly upregulated in the acute phase following TBI and contributes to neuroinflammation and BBB breakdown [16]. IL-6 is produced by glial and endothelial cells and is implicated in leukocyte recruitment, vascular permeability, and edema formation [17]. The observed positive correlation between IL-6 and both S100β (r = 0.583) and GFAP (r = 0.537) reinforces its role as a downstream effector of glial activation and BBB disruption.

Our findings showed a significant increase in MDA levels (5.12 ± 1.03 µmol/L) and concurrent reductions in antioxidant enzymes SOD (1.68 ± 0.42 U/g FW) and catalase (3.45 ± 0.79 U/g FW) in TBI patients (p < 0.001 for all comparisons). MDA, a lipid peroxidation end-product, reflects ROS-mediated damage and is widely recognized as a hallmark of oxidative stress in TBI [18]. Studies by Abdul-Muneer et al. [15] and others have demonstrated that ROS such as superoxide and hydroxyl radicals disrupt cellular membranes and promote neurovascular inflammation via matrix metalloproteinase (MMP) activation [19,20].

The decline in SOD and catalase activities observed in this study supports the view that the endogenous antioxidant system becomes overwhelmed during secondary injury. As reported in previous experimental models, diminished antioxidant enzyme levels contribute to mitochondrial dysfunction, apoptotic signaling, and impaired cerebral perfusion following TBI [21,22]. Our findings agree with these observations and reinforce the mechanistic link between oxidative stress and neuronal/glial injury.

Significant positive correlations between S100β and MDA (r = 0.642) and between GFAP and MDA (r = 0.604), as well as negative correlations between S100β and SOD (r = -0.461) and between GFAP and catalase (r = -0.491), suggest a tightly interwoven relationship between glial damage, inflammation, and oxidative stress. These findings align with the cascade described in the literature, where ROS-mediated oxidative damage exacerbates glial activation, which in turn amplifies cytokine production and compromises the BBB [23].

Furthermore, the upregulation of inflammatory cytokines and oxidative stress-induced enzymatic activity (e.g., NADPH oxidase, iNOS) promotes a self-reinforcing loop of neuroinflammation and oxidative injury, as supported by multiple preclinical studies [24]. The current data substantiate this pathophysiological model.

Our results are consistent with findings from both human and experimental TBI studies. For instance, elevated S100β and GFAP levels have been reported in both serum and cerebrospinal fluid (CSF) shortly after injury and are considered reliable indicators of BBB permeability and glial activation [25]. Similarly, increased MDA and decreased antioxidant enzymes were observed in several experimental TBI models, corroborating the central role of oxidative stress in TBI pathology [21].

However, variations in IL-6 levels across different studies may relate to differences in injury severity, sample timing, and analytical techniques. Some studies also suggest a dual role for IL-6, where low levels may be neuroprotective while excessive production exacerbates inflammation and tissue damage [12]. This complexity underscores the importance of temporal profiling and patient stratification in future studies.

This study has several limitations that should be considered when interpreting the findings. It was conducted at a single center, which may limit the generalizability of the findings. The sample size, though adequate for primary analysis, was not sufficient for detailed subgroup analysis by TBI severity. Additionally, the study did not include serial measurements, limiting insight into the temporal progression of biomarker levels. Potential confounding factors such as comorbidities and medication history were not fully controlled. It is also important to note that elevations in these biomarkers may also occur in conditions other than TBI, which could act as potential confounders. S100β levels, for example, can increase in extracranial trauma, major orthopedic injuries, and systemic inflammatory states due to its expression in adipocytes and chondrocytes [26]. Similarly, IL-6 is a non-specific pro-inflammatory cytokine that rises in sepsis, autoimmune diseases, and even post-surgical states, independent of brain injury [26]. Oxidative stress markers such as MDA and antioxidant enzymes can also be influenced by metabolic disorders, chronic infections, and nutritional deficiencies. Although our study excluded participants with known chronic inflammatory or systemic diseases, the possibility that subclinical conditions may influence the biomarker profile cannot be completely ruled out.

## Conclusions

Overall, the findings of this study reinforce the mechanistic links among oxidative stress, glial injury, and neuroinflammation in TBI. Elevated levels of S100β, GFAP, IL-6, and MDA, along with reduced antioxidant defenses, reflect a pathological cascade consistent with prior literature. These markers offer potential not only for diagnosis and severity assessment but also for monitoring therapeutic interventions targeting oxidative and inflammatory pathways.
